# Unearthing a Cryptic Biosynthetic Gene Cluster for the Piperazic Acid-Bearing Depsipeptide Diperamycin in the Ant-Dweller *Streptomyces* sp. CS113

**DOI:** 10.3390/ijms25042347

**Published:** 2024-02-16

**Authors:** Coral García-Gutiérrez, Ignacio Pérez-Victoria, Ignacio Montero, Jorge Fernández-De la Hoz, Mónica G. Malmierca, Jesús Martín, José A. Salas, Carlos Olano, Fernando Reyes, Carmen Méndez

**Affiliations:** 1 Departamento de Biología Funcional e Instituto Universitario de Oncología del Principado de Asturias (I.U.O.P.A), Universidad de Oviedo, 33006 Oviedo, Spain; coral93@hotmail.es (C.G.-G.); nachomontero@gmail.com (I.M.); jorgefhoz@gmail.com (J.F.-D.l.H.); monicagomal@yahoo.es (M.G.M.); jasalas@uniovi.es (J.A.S.); olanocarlos@uniovi.es (C.O.); 2 Instituto de Investigación Sanitaria de Asturias (ISPA), 33011 Oviedo, Spain; 3 Fundación MEDINA, Centro de Excelencia en Investigación de Medicamentos Innovadores en Andalucía, 18016 Granada, Spain; ignacio.perez-victoria@medinaandalucia.es (I.P.-V.); jesus.martin@medinaandalucia.es (J.M.); fernando.reyes@medinaandalucia.es (F.R.)

**Keywords:** *Streptomyces*, diperamycin, piperazate, depsipeptide, azinothricin, N-N bond, hydrazine, genome mining, secondary metabolites, natural products, NRPS, PKS

## Abstract

Piperazic acid is a cyclic nonproteinogenic amino acid that contains a hydrazine N-N bond formed by a piperazate synthase (KtzT-like). This amino acid, found in bioactive natural products synthesized by non-ribosomal peptide synthetases (NRPSs), confers conformational constraint to peptides, an important feature for their biological activities. Genome mining of *Streptomyces* strains has been revealed as a strategy to identify biosynthetic gene clusters (BGCs) for potentially active compounds. Moreover, the isolation of new strains from underexplored habitats or associated with other organisms has allowed to uncover new BGCs for unknown compounds. The in-house “Carlos Sialer (CS)” strain collection consists of seventy-one *Streptomyces* strains isolated from the cuticle of leaf-cutting ants of the tribe *Attini*. Genomes from twelve of these strains have been sequenced and mined using bioinformatics tools, highlighting their potential to encode secondary metabolites. In this work, we have screened in silico those genomes, using KtzT as a hook to identify BGCs encoding piperazic acid-containing compounds. This resulted in uncovering the new BGC *dpn* in *Streptomyces* sp. CS113, which encodes the biosynthesis of the hybrid polyketide–depsipeptide diperamycin. Analysis of the diperamycin polyketide synthase (PKS) and NRPS reveals their functional similarity to those from the aurantimycin A biosynthetic pathway. Experimental proof linking the *dpn* BGC to its encoded compound was achieved by determining the growth conditions for the expression of the cluster and by inactivating the NRPS encoding gene *dpnS2* and the piperazate synthase gene *dpnZ*. The identity of diperamycin was confirmed by High-Resolution Mass Spectrometry (HRMS) and Nuclear Magnetic Resonance (NMR) and by analysis of the domain composition of modules from the DpnP PKS and DpnS NRPS. The identification of the *dpn* BGC expands the number of BGCs that have been confirmed to encode the relatively scarcely represented BGCs for depsipeptides of the azinothricin family of compounds and will facilitate the generation of new-to-nature analogues by combinatorial biosynthesis.

## 1. Introduction

Functional groups containing nitrogen-nitrogen (N-N) bonds have been identified in more than 200 natural products isolated from different sources [1]. This is a structurally diverse group of compounds that shows a variety of biological activities, including antibiotic, anticancer, anti-inflammatory and analgesic properties, among others [1,2]. This group of compounds features a wide variety of functional groups including azoxy, diazo, hydrazide, hydrazine, nitramine, nitrosamine and heterocyclic motifs [3]. Piperazic acid is a cyclic hydrazine nonproteinogenic amino acid found in peptide compounds synthesized by NRPSs or by hybrid NRPS–PKSs [4]. This amino acid, as well as proline, confers structural rigidity to peptides, which is important for their biological activities. The biosynthetic origin of piperazate remained unknown for several decades until 2012, when Walsh and collaborators identified and characterized KtzI from the kutzneride biosynthetic gene cluster [5]. This is the first enzyme of the pathway that converts L-ornithine into L-*N^5^*-OH-ornithine. More recently, Ryan and colleagues identified KtzT as the piperazate synthase responsible for the formation of the N-N bond in L-*N^5^*-OH-ornithine to generate piperazic acid [6]. Several KtzT homologs have been identified in different actinomycetes and in a few proteobacteria genomes, where they are misannotated as FMN-binding negative transcriptional regulators (PaiB-like) [6,7]. However, piperazate synthases differ from PaiB-like regulators both in their function and amino acid sequences, clustering in different clades and showing characteristic amino acids at specific positions [7]. Several putative BGCs encoding piperazate-containing compounds have been reported, but only in a few cases has their involvement in the biosynthesis of those compounds been confirmed [4]. With some exceptions, most of these clusters contained piperazate biosynthesis-encoding genes [7,8]. 

Bacteria belonging to the genus *Streptomyces* are mainly known for being producers of a high number of secondary metabolites (also known as specialized metabolites), many of which show some kind of bioactivity. In the last two decades, many *Streptomyces* genomes have been sequenced and revealed to contain a high number of BGCs. The development of bioinformatics tools such as the Antibiotic and Secondary Metabolites Analysis Shell (antiSMASH) [9] have allowed for the mining of *Streptomyces* genomes, searching for putative BGCs to ascribe them to specific biosynthetic pathways. Moreover, the isolation of new strains from underexplored habitats or associated with other organisms (insects, plants or sponges) has allowed researchers to uncover new BGCs for unknown compounds [10,11,12]. However, in many cases, definite proof connecting the BGC to the encoded compound has been lacking, either because the putative encoded compound has not been identified in cultures of the producer strain and/or because there is no genetic evidence that links the BGC to the compound. 

The “CS” strain collection is an in-house collection consisting of seventy-one *Streptomyces* strains isolated from the cuticle of leaf-cutting ants belonging to the tribe *Attini*, which were collected in Lambayeque (Perú) [13,14]. Previously, genomes from twelve of these strains have been sequenced by our research group and mined for BGCs using bioinformatics tools such as antiSMASH [9], which brought to light the fact that they harbor between 22 and 44 BGCs, highlighting the potentiality of these strains to synthesize secondary metabolites [13,15]. In previous works from our research group, these strains have been screened to search for BGCs encoding glycosylated or halogenated compounds. This has resulted in the identification of several BGCs encoding glycosylated compounds, including novel ones, such as those for sipanmycins and members of the warkmycin family [13], and a BGC for the novel halogenated compounds colibrimycins [14]. The synthesis of these compounds was achieved by growing the producer strains under standard laboratory conditions, or, when the identified BGCs were silent, awakening their expression was achieved by growing the strains in different culture media and/or by overexpressing cluster-situated regulatory genes [13]. Herein, we report the in silico screening of the twelve sequenced genomes of the “CS” strain collection to identify BGCs encoding piperazic acid-containing compounds, the uncovering of the unknown BGC *dpn* in *Streptomyces* sp. CS113 and the confirmation of its involvement in the biosynthesis of the hybrid polyketide–depsipeptide diperamycin belonging to the azinothricin family of compounds. 

## 2. Results and Discussion

### 2.1. Identification of the Dpn Biosynthetic Gene Cluster

**Figure 1:**
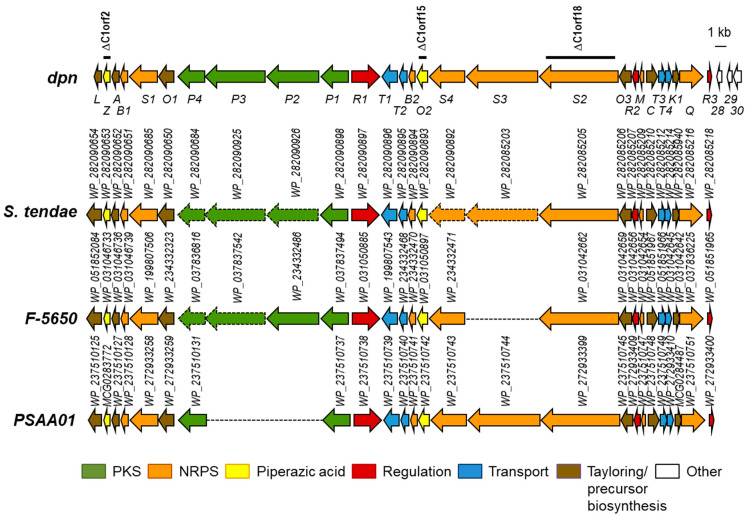
Genetic organization of cluster *dpn* from *Streptomyces* sp. CS113 and comparison to homologous gene clusters in other *Streptomyces* strains. Clusters shown are from *S. tendae*, *Streptomyces* sp. NRRL F-5650 and *Streptomyces* sp. PSAA01. Genes belonging to the BGCs are colored. Other genes are in white. Arrows with dashed lines indicate genes not wholly sequenced. Dashed lines indicate not-assembled DNA regions. Genes are shown to scale. Black bars indicate DNA regions that have been deleted in *Streptomyces* sp. CS113R mutants.

As part of an ongoing investigation by our research group about the potentiality of the CS *Streptomyces* strain collection isolated from ants to synthesize bioactive compounds, we have screened the twelve *Streptomyces* CS genomes previously sequenced [13] to identify BGCs encoding piperazate-containing compounds, using the piperazate synthase KtzT as a hook [6]. Quite recently, a similar approach was carried out using the same probe to screen bacterial genomes in the National Center for Biotechnology Information (NCBI) database, resulting in the identification of the incarnatapeptine BGC [15]. More recently, the use of another strategy based on the Polymerase Chain Reaction (PCR) amplification of *ktzI*- and *ktzT*-homologous genes from the bacterial genomic DNA of strains isolated in different environments has led to the identification of putative BGCs for depsidomycins and lenziamides [8]. By using KtzT as probe against the “CS” genomes in a Basic Local Alignment Search Tool (BLAST) analysis, we could identify a protein similar to KtzT (WP_087803920.1; 54.67% identical amino acids) in *Streptomyces* sp. CS113. This protein contained the highly conserved sequence motif KLSQ (residues 177–181), flanked by methionine (position 176) and glutamic acid (position 182) residues, which are characteristic of piperazate synthases and differentiate them from PaiB-like regulators [7]. Its coding gene was located at cluster 1, identified by antiSMASH version 7.0.0 in the CS113 genome (NZ_KZ195574.1; nucleotides 1 to 84357). This BGC (renamed *dpn*, see below) showed similarity to known depsipeptide BGCs such as those for polyoxypeptin, aurantimycin A or incarnatapeptin/dentigerumycin (37%, 36% and 65% of the genes showed similarity, respectively). These similarities suggested that the *dpn* BGC would correspond to a new BGC, most probably encoding a hybrid polyketide–depsipeptide compound. Further protein BLAST (BLASTP) analyses of each *dpn* gene product allowed us to identify three partially sequenced unknown BGCs in other *Streptomyces* strains (*S. tendae*, *Streptomyces* sp. NRRL F-5650 and *Streptomyces* sp. PSAA01) (Figure 1; Table S1), whose gene products were highly similar to those from CS113, displaying the same gene organization. 

Based on the high synteny among all these clusters, *dpnL* and *dpnR3* are proposed to be the limits of the *dpn* cluster (Figure 1) that would span 66.5 kb and comprise twenty-seven genes. The *dpn* BGC would include genes for the piperazate synthase (*dpnZ*) and for a putative ornithine oxygenase (*dpnO2*) that together would synthesize piperazic acid; genes for Type I PKS (*dpnP1* to *dpnP4*) organized into four modules; genes for NRPS (*dpnS1* to *dpnS4*) organized into six modules; regulatory genes (*dpnR1* to *dpnR3*); genes for transport proteins (*dpnT1* to *dpnT4*); and other genes for tailoring reactions and for the biosynthesis of the precursor building blocks (Table S1; Figure 1). The analysis of NRPS and PKS modules and domains organization (see below) suggested that the *dpn* BGC would encode a piperazic acid-containing hybrid polyketide–hexapeptide compound.

### 2.2. Monitoring Expression of the Dpn Biosynthetic Gene Cluster in Different Culture Media

Many BGCs are silent or marginally expressed under standard laboratory conditions, and changes in media composition can result in an increase in their expression [16]. To monitor the expression of the *dpn* BGC in different culture conditions, a reporter vector pOJ260ind was constructed that utilizes the indigoidine reporter gene *indC* from *S. albus* [17]. Then, the plasmid pOJ260ind-C1 (Table 1) was generated to insert *indC* in the *dpn* BGC downstream of *dpnO3*, and, therefore, to monitor its expression.

**Figure 2:**
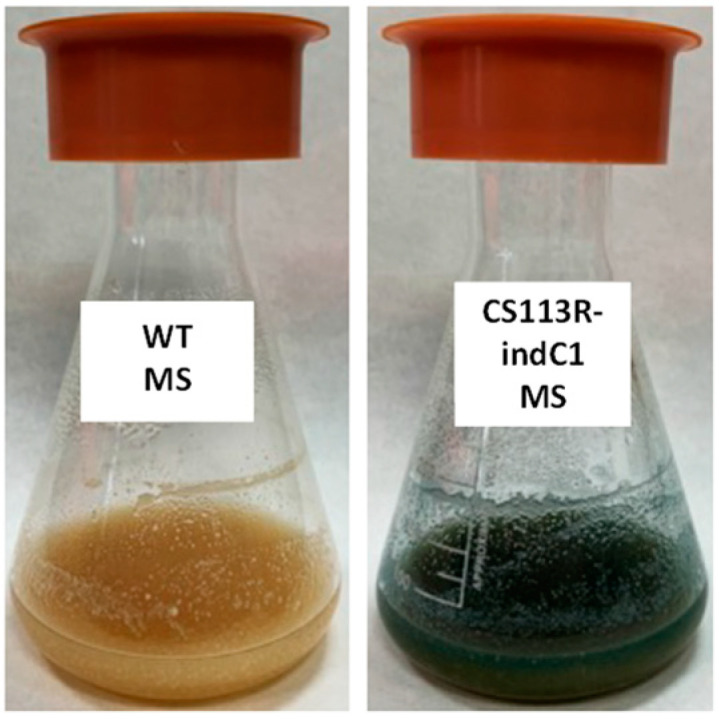
Production of indigoidine by CS113R-indC1 in MS medium. WT: *Streptomyces* sp. CS113R wild type strain.

The genotype of the resultant recombinant strain CS113R-indC1 was confirmed by PCR: using oligonucleotides M13-F and 113C1orf18-indC-comp-R (Table S2), a 2.2 kb DNA fragment was amplified from *Streptomyces* CS113R-indC1 and not from the wild type strain, confirming the insertion of pOJ260ind-C1 at the right position in the *dpn* BGC (see Figure S1 in Supplementary Materials). Afterwards, the expression of the *dpn* BGC by that strain was analyzed in nine different culture media by detecting production of the blue indigoidine pigment. The wild type strain CS113R was used as a control. Only in one of these media (Mannitol Soya flour medium, MS medium) was the production of indigoidine by CS113R-indC1 detected (Figure 2), indicating that this medium could be suitable for the expression of the *dpn* BGC and therefore for identifying its encoded compound. 

### 2.3. Identification and Structural Elucidation of the Dpn-Encoded Compound 

To identify the biosynthesis product of the *dpn* BGC, a mutant in the NRPS gene *dpnS2* was designed to completely block the biosynthesis of compounds directed by this cluster (Figure 1). Using plasmid pHZ-CS113-C1orf18 (Table 1), *dpnS2* was deleted and replaced by an apramycin resistance cassette that was inserted in the same direction of transcription. The genotype of the resultant mutant strain CS113R-ΔC1orf18 was confirmed by PCR using oligonucleotides 113C1orf18-comp-F and 113C1orf18-comp-R (Table S2), which amplified a 1.5 kb DNA fragment corresponding to the apramycin resistance cassette from the mutant and a 8.8 kb from the wild type strain, confirming the replacement of *dpnS2* by the apramycin resistance gene (Figure S2). Further confirmation was obtained using primers NRPSint113_I_RV and NRPSint113_D_FW, which amplified the expected 2.7 kb from the wild type but not from the mutant strain (Figure S2). 

**Figure 3:**
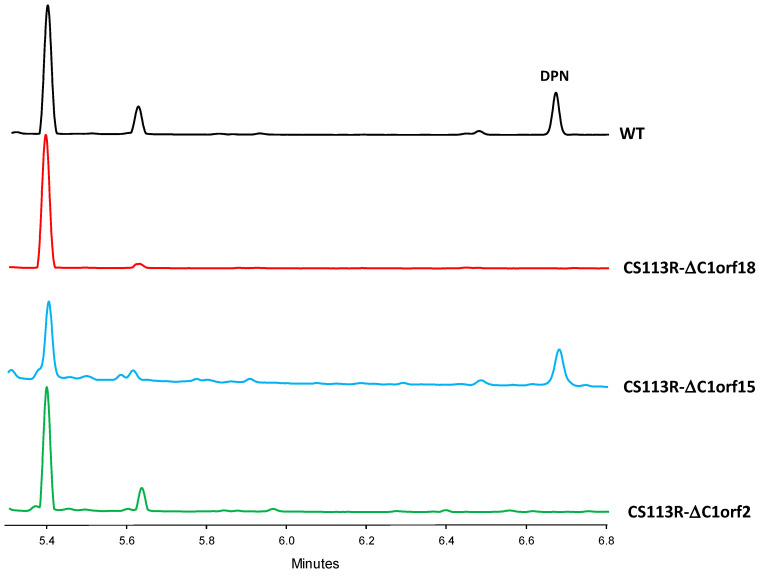
Production of diperamycin by *Streptomyces* sp. CS113R. UPLC chromatograms of ethyl acetate extracts of *Streptomyces* sp. CS113R (WT), and mutants CS113R-Δorf18, CS113R-Δorf15 and CS113R-Δorf2 cultivated in MS medium. DPN: diperamycin.

The CS113R-ΔC1orf18 mutant and the wild type strain CS113R (as control) were cultivated in MS medium. Culture samples were harvested at different times during growth and extracted with different solvents, and extracts were analyzed by Ultra Performance Liquid Chromatography (UPLC) to compare their metabolite profiles. The analysis of chromatograms of ethyl acetate extracts revealed the presence of one compound (peak labelled as DPN in Figure 3) eluting at 6.6 min that was present in the wild type strain CS113R and absent in CS113R-ΔC1orf18 mutant (Figure 3). 

**Figure 4:**
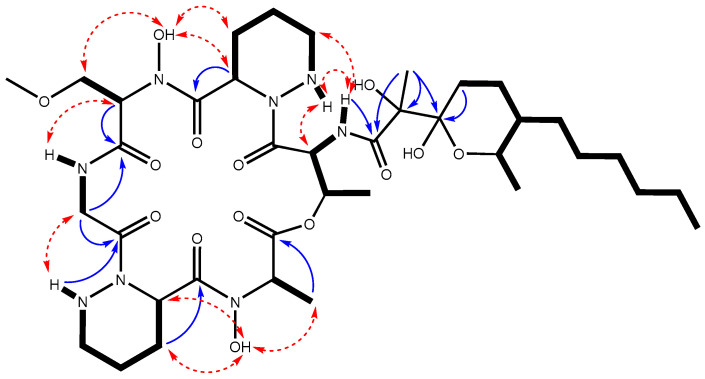
Key COSY and TOCSY correlations (bold bonds) determining the different spin systems of diperamycin. Key NOESY correlations (red dashed double arrows) enabling the determination of the amino acid sequence of the peptidic part of the compound, which, together with the observed key HMBC correlations (blue arrows, H to C), rendered the connectivity of DPN.

After the purification of this compound, further High Performance Liquid Chromatography (HPLC)-HRMS analysis rendered for it a molecular formula of C_38_H_64_N_8_O_14_ based on the observed [M+NH_4_^+^] adduct at an *m*/*z* value of 874.4869 (calculated for C_38_H_68_N_9_O_14_^+^ = 874.4880, Δ = 1.3 ppm). The determined molecular formula matches those of aurantimycin A and diperamycin [18,19], two structurally related members of the azinothricin family of natural products likewise produced by *Streptomyces* strains. The structure of these compounds is in agreement with the molecular size and structural type expected for a compound encoded by the *dpn* BGC. To ascertain whether the target compound corresponded to diperamycin, aurantimycin A or even a novel isobaric compound, a full spectroscopic analysis by NMR, including the acquisition of 1D (^1^H and ^13^C) and 2D (Correlation Spectroscopy, COSY; Total Correlation Spectroscopy, TOCSY; Nuclear Overhauser Effect Spectroscopy, NOESY; Heteronuclear Single Quantum Coherence, HSQC;and Heteronuclear Multiple Bond Correlation, HMBC) NMR spectra (Figures S3–S9), was pursued. Analysis of the ^1^H and HSQC spectra immediately confirmed that the compound belonged to the azinothricin family of natural products [4]. Combining the key observed COSY and TOCSY correlations (Figure 4) allowed the identification of the spin systems of the peptidic and polyketide parts of the compound. The key observed HMBC correlations (Figure 4) established unambiguously the connectivity of all the amino acids and the connectivity of the polyketide moiety (positions 1–14). The two amino acids lacking the amide proton were N-hydroxylated to satisfy the molecular formula and to account for the two sharp exchangeable proton signals observed around 10 ppm. Complete sequencing of the peptide using the HMBC spectrum was hampered, due to the absence of some key correlations. Nevertheless, complementary use of the observed key NOESY correlations (Figure 4) enabled the unambiguous establishment of the peptide sequence and the full compound connectivity, which turned out to correspond to diperamycin (DPN) (Figure 4). Further comparison of our NMR data (Table S3) with those reported for DPN [19] confirmed the identity of the compound, and consequently the BGC was named *dpn*. 

**Figure 5:**
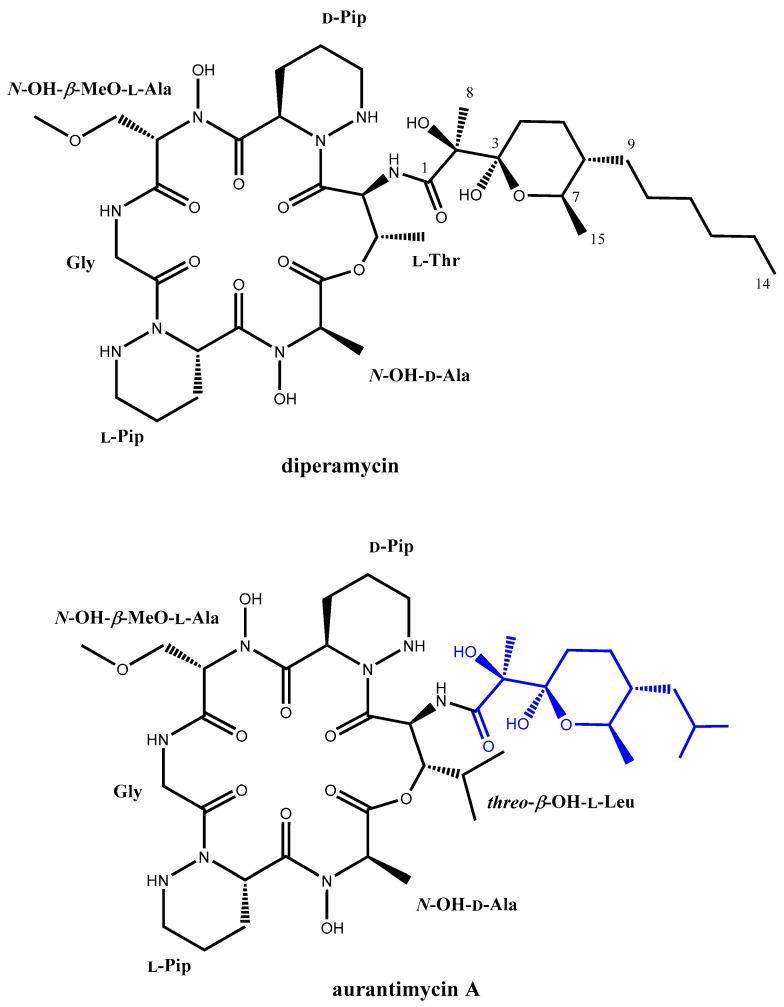
Chemical structure of diperamycin and the structurally related aurantimycin A. The polyketide moiety of aurantimycin A (highlighted in blue) was wrongly sketched with the opposite stereochemistry in the original report describing its discovery.

Although only the planar structure of DPN has been described [19], a comparison of its reported NMR data with those reported for aurantimycin A [18] clearly indicates the same configuration for all the chiral centers in both compounds. Regrettably, the stereochemistry of the polyketide moiety of aurantimycin A was sketched wrongly in the original report describing its discovery, displaying opposite stereochemistry to the one determined by the authors using X-ray crystallography [19]. Unfortunately, such error has been inherited in recent publications that cite the original aurantimycin A report [4,20]. Herein, we take advantage to correct such a mistake for the benefit of the scientific community (Figure 5). Overall, the similar domain composition of PKS and NRPS from the aurantimycin BGC [20] and the *dpn* BGC (see below) ensure identical absolute configurations for the chiral centers in the peptidic and polyketide moieties of both aurantimycin A and DPN (Figure 5). In fact, it has been pointed out in the past that the structural similarity of the different members of the azinothricin family of natural products suggests conserved biosynthetic pathways [4]. Thus, is not surprising that the configuration of the chiral centers in the polyketide moieties of these compounds remains identical [4], considering the amendment of the original aurantimycin configuration herein reported.

### 2.4. Involvement of Piperazic Acid Biosynthesis Genes in Production of Diperamycin

DPN contains two units of piperazic acid (Figure 4). The biosynthesis of this nonproteinogenic amino acid requires two enzymes: an ornithine oxygenase that converts L-ornithine into *N^5^*-OH-L-ornithine, and a piperazate synthase that cyclizes the hydroxylated ornithine to generate piperazic acid [5,6]. The *dpn* BGC contains two genes (*dpnO2* and *dpnZ*) which could fulfill these two roles. To determine their involvement in the biosynthesis of DPN, these two genes were independently mutated (Figure 1) and the metabolite profiles of the resultant mutants were compared to that from the wild type strain (Figure 3). The results were as follows:

- Regarding the mutant in the ornithine monooxygenase-encoding gene *dpnO2*, the mutant CS113R-ΔC1orf15 was generated (Table 1) using pHZ-CS113-C1orf15. Its genotype was confirmed using oligonucleotides 113C1orf15-comp-F and 113C1orf15-comp-R (Table S2), which amplified a 1.5 kb DNA fragment from the mutant while also amplifying a 1.4 kb fragment from the wild type strain, confirming the deletion of *dpnO2* in CS113R-ΔC1orf15 and its replacement by the apramycin resistance gene (Figure S10). The UPLC analysis of culture extracts from the resultant mutant CS113R-ΔC1orf15 (Table 1) revealed that it still produced DPN (Figure 3). This fact suggested that either the putative ornithine hydroxylase DpnO2 was not required for the production of DPN or, alternatively, that there was an additional copy of an ornithine hydroxylase-encoding gene in the CS113R genome. The existence of additional genes encoding ornithine hydroxylase has been described in other *Streptomyces* genomes [7]. In accordance to this, a search for additional ornithine hydroxylase-encoding genes was carried out in the CS113 genome. This resulted in the identification of an additional ornithine hydroxylase gene (WP_087804462) coding for a protein that shares 51% identical amino acids with DpnO2. The existance of this gene would explain why the CS113R-ΔC1orf15 mutant still produced DPN.
- Regarding the mutant in the piperazate synthase-encoding gene *dpnZ*, this gene was delated using plasmid pHZ-CS113-C1orf2 (Table 1). The resultant mutant CS113R-ΔC1orf2 was confirmed using oligonucleotides CS113C1orf2-comp-F and 113C1orf2-comp-R (Table S2), which amplified a 1.5 kb DNA fragment from the mutant strain and a 1 kb fragment from the wild type strain, confirming the replacement of *dpnZ* by the apramycin resistance cassette (Figure S11). The resultant mutant strain CS113R-ΔC1orf2 (Table 1) was cultivated and its metabolite profile was analyzed by UPLC. As observed in Figure 3, this mutant did not produce DPN, confirming that *dpnZ* was essential for DPN biosynthesis as a piperazate synthase-encoding gene, and additionally confirming that DPN is a piperazate-bearing compound. The production of DPN was recovered in CS113R-ΔC1orf2 when *dpnZ* was expressed *in trans* using pSETETc-C1orf2 (Table 1; Figure S12). These and the above-mentioned results confirmed the involvement of *dpn* BGC in the biosynthesis of DPN.

### 2.5. Analysis of Diperamycin Biosynthetic Gene Cluster and Proposed Biosynthesis Pathway

**Figure 6:**
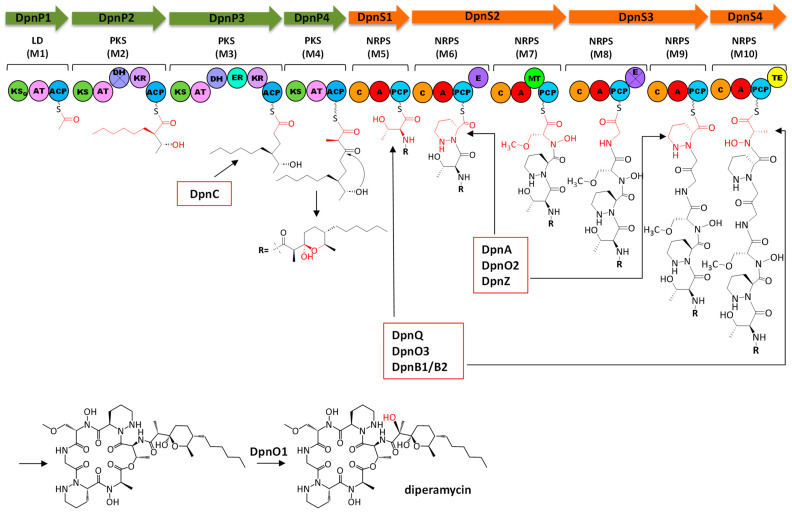
Proposed biosynthetic pathway for diperamycin. PKS and NRPS similar domains are shown in the same color. Crossed domains indicate they are inactive.The biosynthesis would start by synthesizing the C_15_ polyketide chain using the Type I PKS DpnP. This comprises four subunits, each one containing one module (M): DpnP1, which corresponds to the loading module (LM), and DpnP2, DpnP3 and DpnP4, which correspond to the extension modules M2, M3 and M4, respectively. All modules contain the minimal three domains of a modular PKS: a β-ketoacyl synthase (KS), an acyltransferase (AT) and an acyl carrier protein (ACP). Moreover, DpnP2 and DpnP3 have a ketoreductase (KR) and a dehydratase (DH) domain, and DpnP3 has an additional enoyl reductase (ER) domain. All ACPs contain a conserved serine residue for attachment of the phosphopantetheine arm [21]. The KS domain of the extension modules features the catalytic triad C-H-H, essential for the decarboxylative condensation of acyl units during the elongation of the polyketide chain [22]. In the KS domain of the DpnP1 LM, the cysteine residue is replaced by glutamine, constituting a ketosynthase-like decarboxylase domain (KS_Q_) [23]. Analyses of AT domains indicate that all contain the GHSxGE motif around the catalytic serine, that the DpnP1 and DpnP4 ATs contain the amino acid sequence YASH specific for methyl-malonyl- Coenzyme A (CoA) and that DpnP3 contains the amino acid sequence HAFH for malonyl-CoA [24]. The AT in DpnP2 does not contain any of those motifs, suggesting that this AT would recognize neither malonyl-CoA nor methyl-malonyl-CoA, but another acyl-CoA. DPN includes an unusual octanoyl unit in the polyketide chain that would be incorporated by DpnP2. These units are usually derived from hexylmalonyl-CoA [25]. This extender unit is also involved in the biosynthesis of some polyketides such as filipin [26] or cinnabaramide A [27]. A comparison between the AT from DpnP2 and the corresponding ATs from PteA5 (filipin) and CinA (cinnabaramide A) highlights the amino acid sequence AGHS in DpnP2, PteA5 and CinA, which could be a specific motif for hexylmalonyl-CoA. The KR domains of DpnP2 and DpnP3 contain the Nicotinamide Adenine Dinucleotide Phosphate (NADPH)-binding site and the catalytic triad K-S-Y. They can be classified into the KR B1 type based on the presence of the amino acid sequence LDD and the absence of a proline residue next to the catalytic tyrosine [24,28]. According to this, the configurations of hydroxy groups in the resulting intermediates would be (2R,3R) 3-hydroxy-2-methylacyl [24,28,29]. The DH domain of DpnP3 contains the catalytic histidine in the HxxxGxxxxP motif and the invariant tyrosine in the amino acid sequence YGP [24], while the DH domain of DpnP2 only preserves the first motif, which suggests that this DH domain is inactive. The ER domain of DpnP3 contains the NADPH-binding site and a valine residue at position 52′, which suggests the formation of a (*2R*) product [29,30]. Therefore, the domain composition of modules from DpnP PKS correlates well with the structure of the C_15_ polyketide chain of DPN. The only disagreement is that, according to the structure of DPN, the biosynthesis of the polyketide moiety would start with acetate, while the AT from the LM DpnP1 would specify for methyl-malonate-CoA. Curiously enough, this discrepancy also occurs in aurantimycin biosynthesis [18,20]. While the aurantimycin compound contains an acetate starter unit, after performing an analysis of the AT domain from the LM ArtO encoded by the aurantimycin BGC (*art*), it was revealed that it would also specify for methyl-malonyl-CoA.

Based on the chemical structure of DPN and on bioinformatics analyses of the *dpn* BGC, a biosynthetic pathway for DPN is proposed (Figure 6). 

Once the lipophilic polyketide chain of DPN is synthesized, this will be delivered to a NRPS for the biosynthesis of the hexapeptide chain. The *dpn* BGC contains eight NRPS related genes: *dpnS1* to *dpnS4*, encoding NRPS enzymes that would constitute the NRPS core for the biosynthesis of the DPN peptidyl chain; *dpnM*, for a MbtH-like protein that would collaborate with NRPS DpnS in the biosynthesis of the peptidyl chain; *dpnQ*, for a didomain NRPS; and two thioesterase (TE) genes (*dpnB1* and *dpnB2*). The DpnS NRPS would consist of four subunits organized into six modules: DpnS1 (M5), DpnS2 (M6 and M7), DpnS3 (M8 and M9) and DpnS4 (M10) (Figure 6). All these modules contain the minimal set of domains for a NRPS: a condensation (C), an adenylation (A) and a peptidyl carrier protein (PCP) domain. In addition, M6 and M8 have an epimerase (E) domain, M7 a methyltransferase domain (MT) and M10 a TE domain. All C domains have the conserved HHxxxDG motif [31], and belong to different subtypes [32]: the C domain from M5 corresponds to the starter domain (C_S_) that would acylate the first amino acid of the peptide chain with the C_15_ polyketide chain; C domains from M6, M8 and M10 are ^L^C_L_ type domains, which would catalyze the condensation of two L-amino acids; and those C domains from M7 and M9 constitute ^D^C_L_ domains that would perform condensation between a D-aminoacyl/peptidyl-PCP donor and a L-aminoacyl-PCP acceptor. All PCP domains contain the serine residue within the conserved motif GGXS for the attachment of 4′-phosphopantetheinyl [33]. Analysis of the substrate specificity-conferring codes of A domains [34,35] from the different modules indicates that those from M6 (DVFSVASY) and M9 (DVFTVAAY) specify L-piperazic acid, and can be classified within code 1 and code 2, respectively, of this type of A domains [36]; the A domains from M7 (DVWHFSLV) and M8 (DILQVGWV) fit well with A domains for L-serine and glycine, respectively, and those from M10 (DMTQVGWV) could specify L-alanine (50% of amino acid residues show similarity). Noticeably, two of these amino acids (L-alanine and L-serine) are *N*-hydroxylated. These hydroxylations usually occur while the amino acid is loaded onto a stand-alone NRPS module, and the modified amino acid is then recognized by the A domain of the NRPS assembly line [37]. In this sense, M7 and M10 A domains would rather specify for *N*-hydroxy-L-serine and *N*-hydroxy-L-alanine instead of L-serine and L-alanine. A similar situation would occur during aurantimycin and polyoxypeptin biosynthesis [20,38]. In these cases, it has been proposed that hydroxylated amino acids were incorporated along their biosynthetic pathways, while the predicted specificities of those A domains corresponded to their non-hydroxylated amino acids. On the other hand, the *N*-hydroxy-L-serine residue putatively incorporated by M7 is further *O*-methylated. It is proposed that the MT domain present in that module would be responsible for this methylation event. According to the substrate specificity-conferring code, the A domain from M5 would specify for L-valine but, curiously enough, the structure of DPN includes a threonine at that position. This kind of disagreement is also found in the biosynthesis of other cyclodepsipeptides of the azinothricin family such as aurantimycin and polyoxypeptin; in both cases, the specificity-conferring code of the first NRPS module (ArtT and PlyX) corresponds to valine, while the actual incorporated amino acid is 3-hydroxyleucine [20,38]. With this exception, there is a rather good correlation between the specificity-conferring codes of the adenylation domains of NRPS DpnS and the structure of the peptide chain of DPN. Concerning the E domains in M6 and M8, the first one contains the conserved motif HHxxxDG and would be responsible for the conversion of L-piperazic acid to its D form; however, the latter does not have that motif, which suggests that it could be inactive. Nevertheless, the requirement of this E domain in M8 is otherwise unnecessary, since it would affect a glycine residue. Release by cyclization of the full-length DPN polyketide–peptide chain would be carried out by the TE domain in M10, which contains a serine residue within the conserved motif GxS [37]. This domain must additionally have epimerase activity to account for the D-configuration of the N-OH-Ala residue (configuration derived for the D-configuration determined for this amino acid in aurantimycin A) [18]. Such additional epimerase activity of the TE domain has been recently discovered in the biosynthesis of the β-lactam nocardicin A [39], and in the release of the NRPS assembly line by cyclization, as recently described for the depsipeptide skyllamycin A [40]. In this sense, the TE domains of DpnS4 and ArtH from the *dpn* and *art* BGCs show similarity to NocB-TE from the nocardicin A BGC [39] (sharing 24% and 36% identical amino acids, respectively) and Sky31-TE from the skyllamycin A BGC [41] (sharing 29% and 30% identical amino acids, respectively). Moreover, the DpnS4-TE and ArtH-TE contain the catalytic triad S-D-H that includes the histidine residue involved in epimerization [39,40], which suggests the dual epimerization and cyclization function of these TE domains, representing another example of such a role of TE domains. Release and cyclization of the peptide chain would generate a non-hydroxylated DPN intermediate that, after hydroxylation, would lead to the formation of the final DPN compound. This final step could be carried out by the FAD-dependent monooxygenase DpnO1 encoded by the cluster (Figure 6). 

The analysis of the Dpn PKS and NRPS enzymes has revealed the functional similarity between these enzymes and those encoded by the *art* BGC [20]. In both cases, the PKS and NRPS enzymes consist of the same number of modules that contain the same type of domains, which assure identical absolute configuration for the chiral centers in the peptidic and polyketide moieties, as stated above. This similarity extends to the existence of inactive domains in both clusters. Thus, the DpnP2 PKS module contains an inactive DH domain, and after carrying out an analysis of the equivalent module ArtP from the *art* cluster it was revealed that this DH domain is also inactive, lacking the two conserved motifs [24]. Moreover, as it occurs in DpnP2, the KR domain of ArtP also belongs to the B1 type, and therefore the configurations of hydroxy groups in the resultant intermediates are identical [24,29,30]. Also, the DpnS3 NRPS contains an inactive E domain, and analysis of the equivalent E domain in ArpG NRPS shows that is also inactive. Therefore, the polyketide–peptide chains synthesized by the Dpn and Art pathways are nearly identical. Differences between both chains derive from the second extender unit recognized by each PKS (hexylmalonyl-CoA in Dpn, and 2-(2-methylpropyl) malonyl-CoA in Art), and from the first amino acid incorporated by each NRPS (L-threonine in Dpn and *threo*-*β*-OH-L-Leucine in Art).

The *dpn* BGC contains several genes that could be involved in the biosynthesis of different precursors of DPN. The DpnP PKS synthesizes the polyketide moiety from two units of malonyl-CoA, one methylmalonyl-CoA and one hexylmalonyl-CoA. This last one is an unusual extender unit that would derive from 2-octenoyl-CoA by the action of a crotonyl-CoA carboxylase/reductase (CCR) [25]. The *dpn* BGC contains a CCR *dpnC* encoding gene that could fulfil this activity (Figure 6). On the other hand, the peptidyl moiety of DPN contains four nonproteinogenic amino acids: two L-piperazic acids, one *N*-hydroxy-L-serine and one *N*-hydroxy-L-alanine. As mentioned above, the biosynthesis of L-piperazic acid requires hydroxylation of L-ornithine followed by the cyclization of the resultant L-*N^5^*-OH-ornithine [5,6]. Similarly to the way it occurs in other BGCs encoding piperazic acid-bearing peptides [8], the *dpn* BGC contains two genes encoding the DpnO2 ornithine *N*-monooxygenase and the DpnZ diperazate synthase for the biosynthesis of L-piperazic acid (Figure 6). Noticeably, in the *art* BGC from *Streptomyces aurantiacus* JA 4570 and the polyoxypeptin A BGC from *Streptomyces* sp. MK498-98 F14, those two genes have not been identified within the clusters [6,20]. Moreover, the *dpn* BGC contains an amidinotransferase *dpnA* encoding gene that could be involved in the biosynthesis of L-ornithine from L-arginine (Figure 6). The *dpn* BGC includes several genes encoding stand-alone NRPS proteins: a dimodular (A-PCP) NRPS DpnQ and two TE domains (DpnB1 and DpnB2). These dissociated proteins are usually involved in the biosynthesis of nonproteinogenic amino acids, which are synthesized tethered to a PCP [37,42]. The two hydroxylated amino acids *N*-hydroxy-L-serine and *N*-hydroxy-L-alanine are probably synthesized using those proteins (Figure 6): L-serine/L-alanine would be recognized by the A domain of DpnQ and then transferred to its PCP domain. Then, both amino acids would be *N*-hydroxylated by DpnO3. This is a NAD(P)/Flavin Adenine Dinucleotide (FAD)-dependent oxidoreductase that shares 74% identical amino acids with PopE that has been proposed to *N*-hydroxylate glycine and L-alanine, during the biosynthesis of polyoxyperuin [43]. It also shares 54% identical amino acids with LrgO, which is involved in the oxidation of the amine group of the largimycin hybrid peptide–polyketide chain before its offloading [44]. Release of the resulting hydroxylated amino acids would be carried out by TE DpnB1/DpnB2. 

## 3. Materials and Methods

### 3.1. Strains and Culture Conditions, Plasmids and DNA Manipulations

*Streptomyces* sp. CS113R was used as a source of DNA, and for gene replacement and gene expression experiments [45]. This strain was generated from CS113 by selecting spontaneous rifampicin resistance colonies to increase secondary metabolites production. *Escherichia coli* DH10B (Invitrogen, Waltham, MA, USA) was used as cloning host for plasmid propagation and *E. coli* ET12567/pUB307 [46] for conjugation experiments between *E. coli* and *Streptomyces*. MS medium was used for sporulation and for diperamycin production, using a two-step culture method [47,48]. When required, antibiotics were added to culture media at the following final concentrations: nalidixic acid (25 µg/mL), apramycin (25 µg/mL), and thiostrepton (50 µg/mL). 

### 3.2. DNA Manipulations and Plasmid Vectors

DNA manipulations, transformations and intergeneric conjugations were performed according to standard procedures for *E. coli* [49] and *Streptomyces* [46]. DNA amplification by PCR was carried out with Herculase II (Stratagene, San Diego, CA, USA) and using 5% dimethyl-sulfoxide (DMSO). Amplicons were confirmed by DNA sequencing. BLAST [50], antiSMASH 7.0 [9], PKS/NRPS Analysis Web-site [51] and SBSPKSv2 [52] were used for DNA and protein sequences analyses. The following plasmid vectors were used along this work: pUO9090 pHZ1358 [53], pOJ260 [54], pCR-BLUNT (Invitrogen) and pSETETc [55]. A reporter vector, pOJ260ind, was constructed to monitor the expression of *Streptoyces* genes, by cloning the indigoidine biosynthesis NRPS *sshg_00313*-encoding gene (*indC*) from *Streptomyces albus* J1074 [17], into the suicide plasmid pOJ260, as follows: a 3.97 kb PCR fragment containing the start and end codons of *indC* was amplified using the oligo primers C9C2 and C9C1 (Table S2), cloned into pCR-BLUNT, and recued as a fragment flanked by EcoRI sitesto be subcloned in the right orientation into pOJ260, previously digested with EcoRI. 

### 3.3. Construction of pOJ260ind-C1 

This plasmid was constructed for monitoring the expression of *dpn* BGC. A 2.07 kb DNA fragment containing *dpnO3* and *dpnR2* was amplified using the oligonucleotides 113C1orf18-indC-F and 113C1orf18-indC-R (Table S2), digested with XbaI and EcoRV and subcloned into the same sites of pOJ260ind. 

### 3.4. Construction of pSETETc-C1orf2 by Gibson Assembly

This plasmid was used to complement mutant CS113R-ΔC1orf2 and was constructed using the Gibson assembly method [56]. A 1.02 kb DNA fragment was amplified using the primers 113C1orf2-OE-Tc-F and 113C1-orf2-OE-Tc-R (Table S2). This fragment was Gibson assembled with pSETETc previously digested with BamHI and EcoRI. 

### 3.5. Generation of Dpn Mutants

Several independent mutants were generated through gene replacement, by deleting specific *dpn* genes and replacing them by an apramycin resistance cassette (Figure 1, Table 1). The genotypes of the resultant mutant strains were confirmed by PCR and by sequencing the resultant amplicons. To this end, several plasmids were constructed (Table 1) using the Gibson assembly method, following a three-step procedure: (i) amplification of a downstream DNA fragment (Fragment A) containing the 3′-end of the gene to be deleted, a sequence pairing the 3′-end of the apramycin resistance gene *aac(3)IV* from pUO9090 and a sequencing pairing the XbaI site region of pHZ1358; (ii) amplification of an upstream DNA fragment (Fragment B) containing the 5′-end of the gene to be delated, a sequence pairing the 5′-end of the apramycin resistance cassette and a sequence pairing the NheI site region of pHZ1358; (iii) amplification of the apramycin resistance cassette from pUO9090 using oligonucleotides ApraGib-F and ApraGib-R (Table S2). Then, these three fragments and plasmid pHZ1358, previously digested with XbaI and NheI, were assembled using the Gibson Assembly kit (New England BioLabs, Ipswich, MA, USA). The following specific A and B fragments were amplified to construct the different plasmids:

- pHZ-CS113-C1orf2. This plasmid was used to delete *dpnZ*, generating the mutant CS113R-ΔC1orf2. Fragment A (1.16 kb), containing *dpnL* and the 3′-end of *dpnZ*, was amplified using the oligonucleotides 113C1orf2A-F and 113C1orf2-R (Table S2). Fragment B (1.56 kb), containing the 5′-end of *dpnZ*, *dpnA* and the 3′-end of *dpnB1*, was amplified using the oligonucleotides 113C1orf2B-F and 113C1orf2B-R (Table S2).
- pHZ-CS113-C1orf15. This plasmid was used to delete *dpnO2*, generating the mutant CS113R-ΔC1orf15. Fragment A (1.84 kb), containing *dpnT2*, *dpnB2* and the 3′-end of *dpnO2*, was amplified using the oligonucleotides 113C1orf15A-F and 113C1orf15A-R (Table S2). Fragment B (1.85 kb), containing the 5′-end of *dpnO2* and the 3′-end of *dpnS4*, was amplified using the oligonucleotides 113C1orf15B-F and 113C1orf15B-R (Table S2).
- pHZ-CS113-C1orf18. This plasmid was used to delete *dpnS2*, generating the mutant CS113R-ΔC1orf18. Fragment A (2.01 kb), containing the 5′-end of *dpnS3* and the 3′-end of *dpnS2*, was amplified using the oligonucleotides 113C1orf18A-F and 113C1orf18A-R (Table S2). Fragment B (2.09 kb), containing the 5′-end of *dpnS2*, *dpnO3* and the 3′-end of *dpnR2*, was amplified using the oligonucleotides 113C1orf18B-F and 113C1orf18B-R (Table S2).

### 3.6. Extraction, Ultra-Performance Liquid Chromatography Analysis and Purification of Diperamycin

Strains were grown in a two-step culture method as previously described in [47]. Culture samples of 1 mL were extracted with an equal volume of ethyl acetate (or ethyl acetate + 1% formic acid). Organic extracts were dried under vacuum, and residues were dissolved in 60 µL of methanol. The production of DPN was monitored at 3, 5 and 7 days of incubation at 30 °C, and analyzed by reversed-phase chromatography on Acquity UPLC equipment with a Etlylene Bridged Hybrid (BEH) C18 column (1.7 mm, 2.1 × 100 mm; Waters, Milford, MA, USA) with acetonitrile and 0.1% trifluoroacetic acid (TFA) in water as an eluent. Samples were eluted with 10% acetonitrile for 1 min, followed by a linear gradient from 10 to 99% acetonitrile over 7 min at a flow rate of 0.5 mL/min and a column temperature of 35 °C. Detection and spectral characterization of peaks were carried out with a photodiode array (PDA) detector and Empower 3 software (Waters, Milford, MA, USA). For the purpose of DPN purification, the wild type strain CS113R was grown as mentioned above, but using forty 250 mL Erlenmeyer flasks, each containing 50 mL of MS medium, in the production step. After 5 days of incubation, cultures were extracted with an equal volume of ethyl acetate plus formic acid, the organic extract was dried down under vacuum and residues were dissolved in a small volume of methanol. The DPN was finally purified by preparative HPLC using a SunFire C18 column (10 mm, 10 × 150 mm, Waters) at a flow of 5 mL/min, with the following mixtures of acetonitrile and 0.1% TFA in water: 45% of acetonitrile for 15 min; 45% to 100% of acetonitrile for 7 min; and 100% to 45% of acetonitrile for 5 min. The purification procedure afforded 1.1.mg of DPN.

### 3.7. Structural Elucidation of Diperamycin

Structural elucidation of DNP was carried out by Electrospray Ionization Time-of-Flight (ESI-TOF ) high-resolution mass spectrometry and NMR spectroscopy. HRMS spectra were collected from Liquid Chromatography (LC)-Diode Array Detector (DAD)-HRMS analyses using an Agilent 1200 Rapid Resolution HPLC system equipped with a SB-C8 column (2.1 × 30 mm, Zorbax, Agilent, Santa Clara, CA, USA) and coupled to a Bruker maXis mass spectrometer. Chromatographic and ionization conditions were identical to those previously described [57]. The diperamycin Ultraviolet (UV)/vis (DAD) spectrum was also collected in the same chromatographic analyses. NMR spectra were registered in Dimethyl Sulfoxide (DMSO)-*d_6_* at 24 °C on a Bruker AVANCE III-500 (500 MHz and 125 MHz for ^1^H and ^13^C NMR, respectively) equipped with a 1.7 mm TCI MicroCryoProbe^TM^, using the residual solvent signal as internal reference (δ_H_ 2.51 and δ_C_ 40.0 for DMSO-*d_6_*). The molecular formula obtained from the experimental accurate mass combined with the analysis of the NMR spectra rendered the DPN chemical structure.

## 4. Conclusions

We have screened in silico *Streptomyces* genomes from the “CS” strain collection, using the piperazate synthase KtzT as a hook, to explore the potential of these strains to produce piperazate-containing compounds. This has led to the identification of the unknown *dpn* BGC in *Streptomyces* sp. CS113 that encodes the cyclic polyketide–hexadepsipeptide diperamycin. Experimental proof linking the *dpn* BGC to its encoded compound was achieved by determining the growth conditions for the expression of the cluster and by generating mutants in specific genes of the cluster. Moreover, we have shown the functional similarity of diperamycin and aurantimycin A PKS and NRPS enzymes, which is reflected in the same configuration for all chiral centers in both compounds. Analysis of the diperamycin biosynthetic gene cluster has led us to propose an amino acid motif for the unusual hexylmalonyl-CoA extender unit in AT domains of PKS. Future work will be required to confirm the specificity of this motif for this acyl-CoA unit. The discovery of the *dpn* BGC expands the number of BGCs and their encoding compounds identified in this strain collection, confirming their genetic capability to encode bioactive compounds. Moreover, the identification of *dpn* BGC increase the number of BGCs encoding depsipeptides of the azinothricin family and broadens the possibility of generating novel potentially active polyketide–depsipeptide compounds by combinatorial biosynthesis, mainly using those *dpn* genes that differentiate the biosynthesis of diperamycin. Further experiments will be required to confirm the proposed functions for those genes, the flexibility of their encoded enzymes to use different substrates and, therefore, their usefulness to generate novel compounds. 

## Figures and Tables

**Table 1:** Strains generated in this study.

Mutant Strain	Plasmid	Deleted Genes
CS113R-ΔC1orf2	pHZ-CS113-C1orf2	*dpnZ*
CS113R-ΔC1orf15	pHZ-CS113-C1orf15	*dpnO2*
CS113R-ΔC1orf18	pHZ-CS113-C1orf18	*dpnS2*
**Recombinant Strain**	**Plasmid**	**Expressed Genes**
CS113R-indC1	pOJ260ind-C1	*indC*
ΔC1orf2 (pSETETc-C1orf2)	pSETETc-C1orf2	*dpnZ*

## Data Availability

The data presented in this study are available in the article and in the Supplementary Materials.

## Supplementary Materials

The supporting information can be downloaded at https://www.mdpi.com/article/10.3390/ijms25042347/s1.
