# Supplementary material for: Unearthing a Cryptic Biosynthetic Gene Cluster for the Piperazic Acid-Bearing Depsipeptide Diperamycin in the Ant-Dweller Streptomyces sp. CS113
Source: Int J Mol Sci. 2024 Feb 16;25(4):2347. doi: 10.3390/ijms25042347 (PMC10888640; doi:10.3390/ijms25042347)
Supplement: Supplementary file 1 [file ijms-25-02347-s001.zip › ijms-2843085-supplementary.pdf]

SUPPLEMENTARY MATERIAL

Unearthing a Cryptic Biosynthetic Gene Cluster for the Piperazic Acid-Bearing Depsipeptide Diperamycin in the Ant-dweller *Streptomyces* sp. CS113

Coral García-Gutiérrez [1,2], Ignacio Pérez-Victoria [3], Ignacio Montero[1,2], Jorge Fernández-De la Hoz,[1] Mónica G. Malmierca [1], Jesús Martín [3], José A. Salas,[1,2] Carlos Olano [1,2], Fernando Reyes [3], and Carmen Méndez [1,2,*]

[1] Departamento de Biología Funcional e Instituto Universitario de Oncología del Principado de Asturias (I.U.O.P.A), Universidad de Oviedo, 33006 Oviedo, Spain

[2] Instituto de Investigación Sanitaria de Asturias (ISPA), 33011 Oviedo, Spain

[3] Fundación MEDINA, Centro de Excelencia en Investigación de Medicamentos Innovadores en Andalucía, 18016 Granada, Spain

* Correspondence: cmendezf@uniovi.es

Table S1. Predicted functions of gene products from the *Streptomyces* sp. CS113 diperamycin biosynthesis gene cluster (*dpn*)

Gene product	aa	Predicted function	*Streptomyces tendae*	*Streptomyces* sp. NRRL F-5650	*Streptomyces* sp. PSAA01
DpnL	286	Fatty acyl-AMP ligase	WP_282090654 (97.37)	WP_051852084 (92.48)	WP_237510125 (86.47)
DpnZ	214	Piperazate synthase	WP_282090653 (95.77)	WP_031046733 (88.32)	MCG0283772 (84.11)
DpnA	266	Amidinotransferase	WP_282090652 (98.5)	WP_031046736 (93.98)	WP_237510127 (88.68)
DpnB1	261	Thioesterase	WP_282090651 (94.25)	WP_031046739 (91.94)	WP_237510128 (84.12)
DpnS1	1081	NRPS	WP_282090685 (95.93)	WP_199807506 (90.79)	WP_272933258 (82.69)
DpnO1	517	FAD-dependent monooxygenase	WP_282090650 (98.44)	WP_234332323 (97.29)	WP_272933259 (92.0)
DpnP4	1045	Type I PKS	WP_282090684 (ND)	WP_037836816 (ND)	WP_237510131 (84.3)
DpnP3	2189	Type I PKS	WP_282090925 (ND)	WP_037837542 (ND)	NI
DpnP2	1871	Type I PKS	WP_282090926 (ND)	WP_234332486 (93.57)	NI
DpnP1	1036	Type I PKS	WP_282090898 (93.39)	WP_037837494 (92.95)	WP_237510737 (83.51)
DpnR1	1047	BTAD domain-containing transcriptional regulator	WP_282090897 (97.13)	WP_031050885 (95.8)	WP_237510738 (87.28)
DpnT1	551	Putative exporter	WP_282090896 (97.51)	WP_199807543 (94.83)	WP_237510739 (86.21)
DpnT2	323	ABC transporter ATP-binding protein	WP_282090895 (99.04)	WP_234332468 (96.59)	WP-237510740 (85.54)
DpnB2	238	Thioesterase	WP_282090894 (98.74)	WP_234332470 (98.32)	WP_237510741 (80.67)
DpnO2	435	Ornithine N-monooxygenase	WP_282090893 (98.39)	WP_031050897 (97.24)	WP_237510742 (87.13)
DpnS4	1266	NRPS	WP_282090892 (ND)	WP_234332471 (92.05)	WP_237510743 (88.39)
DpnS3	2569	NRPS	WP_282085203 (ND)	NI	WP_237510744 (83.91)
DpnS2	2888	NRPS	WP_282085205 (97.33)	WP_031042662 (94.91)	WP_272933399 (87.08)
DpnO3	395	NAD(P)/FAD-dependent oxidoreductase	WP_282085206 (99.49)	WP_031042659 (96.46)	WP_237510745 (92.41)
DpnR2	227	LmbU family transcriptional regulator	WP_282085207 (96.04)	WP_031042656 (94.59)	WP_272933409 (83.48)
DpnM	72	MbtH family protein	WP_282085209 (98.57)	WP_031042654 (98.61)	WP_237510747 (87.50)
DpnC	460	Crotonyl-CoA carboxylase/reductase	WP_282085210 (98.91)	WP_051851967 (97.17)	WP_237510748 (92.39)
DpnT3	273	ABC transporter ATP-binding protein	WP_282085212 (98.90)	WP_051851966 (96.34)	WP_237510749 (87.50)
DpnT4	253	ABC transporter permease	WP_282085214 (99.21)	WP_031042645 (98.81)	WP_272933410 (93.36)
DpnK1	274	SDR family oxidoreductase	WP_282085940 (99.64)	WP_031042642 (97.81)	MCG0284487 (90.51)
DpnQ	902	NRPS	WP_282085216 (96.78)	WP_037836225 (91.01)	WP_237510751 (81.58)
DpnR3	205	LmbU family transcriptional regulator	WP_282085218 (98.86)	WP_051851965 (96.59)	WP_272933400 (87.50)

Numbers between brackets indicate percentage of identical amino acids to the corresponded Dpn protein. ND, not determined. NI, not identified. aa, number of amino acids of Dpn proteins.

Table S2. Oligonucleotides used for PCR amplification

PRIMER	SEQUENCE 5'-3'*
C9C2	GATATCATGCCGTCTCGCACTCCC
C9C1	GAATTCCTAGGCGAAGAGGTCCAAGG
113C1orf18-indC-F	AAAA**TCTAGA**GAGCCGATACCAGATGGAG
113C1orf18-indC-R	AAA**AGATAT**CGCTGTCTCCAAATCGGCA
113C1orf18-indC-comp-R	CGTTGTAGGTCGGTGACG
M13-F	GTAAAACGACGGCCAG
113C1orf18A-F	CTTGACATTGGGGAATTGACGTCGACATGAAGGTCACG
113C1orf18A-R	CGGGGACCTGCAGGTCGACTCAGGAAGTTCGCCAGTGC
113C1orf18B-F	CCGCCGAAAGTTCCTCGAAGGCGAGCTGACCAATTCGTC
113C1orf18B-R	GGCATCAGTTACCGTGAGCGTACGGAGTACCTCGTGC
113C1orf18-comp-F	TGATCGACCGGTACTTCTCG
113C1orf18-comp-R	CAACGGGATCATCGACGCTC
NRPSint113_I_RV	CGAGGACGTCGATGG
NRPSint113_D_FW	GCACGACAGGTGTTC
113C1orf2A-F	CTTGACATTGGGGAATTGACGGCAACAGTCCTTCGCCGACAG
113C1orf2A-R	CGGGGACCTGCAGGTCGACTCACGGGCACACAGTTCGTAG
113C1orf2B-F	CCGCCGAAAGTTCCTCGAAGGAGAACGACGACTTGATGGC
113C1orf2B-R	GGCATCAGTTACCGTGAGCGGACTGCGAGTGATCAGTTC
113C1orf2-comp-F	GGATCCGCACTGACGCTGGACAACAG
113C1orf2-comp-R	GGATCCTGGGCAGATCACGAGAGTGC
113C1orf15A-F	CTTGACATTGGGGAATTGACGAGTGGGGAGATCGTGGAATC
113C1orf15A-R	CGGGGACCTGCAGGTCGACTTGACGCATGAACAGGTCCTC
113C1orf15B-F	CCGCCGAAAGTTCCTCGAAGCGGCTTTCTGGCAGATGTTG
113C1orf15B-R	GGCATCAGTTACCGTGAGCGGGACCGAAACCGATACCG
113C1orf15-comp-F	GGATCCACGCCACACATGACGAAATG
113C1orf15-comp-R	GGATCCTCAACTCCGATCACTGGTCC
113C1orf2-OE-Tc-F	ACAATCGTGCCGGTTGGTAGGCACTGACGCTGGACAACAG
113C1orf2-OE-Tc-R	ACAGCTATGACATGATTACGTGGGCAGATCACGAGAGTGC
ApraGib-F	GCTCACGGTAACTGATGCC
ApraGib-R	CGTCAATTCCCCAATGTCAAG

* Restriction sites are in bold; nucleotide sequences for Gibson assembly of DNA are underlined.

Table S3. NMR data of diperamycin in DMSO-d_6 (500 MHz, 24 °C)

Position	δ_C, type	δ_H, mult. (J in Hz)
**L-Thr**		
CO	167.7, C	
Cα	48.2, CH	5.56, br s
Cβ	71.4, CH	4.76, br s
Cγ	15.9, CH_3	1.24, d (6.2)
NH		8.12, d (9.0)
**D-Pip**		
CO	174.5, C	
Cα	48.4, CH	5.33, dd (6.7, 2.8)
Cβ	24.3, CH_2	1.84, m (ov.)<br>2.07, m (ov.)
Cγ	20.6, CH_2	1.51, m (ov.)
Cδ	46.3, CH_2	2.67, m (ov.)<br>3.02, br d (11.5)
NH		5.00, br d (11.9)
***N*-OH-*β*-MeO-L-Ala**		
CO	167.2, C	
Cα	57.3, CH	5.14, dd (8.1, 5.5)
Cβ	67.1, CH_2	3.70, dd (10.8, 5.5)<br>3.75, dd (10.8, 8.1)
CH_3O	57.9, CH_3	3.26, s
NOH		10.29, s
**Gly**		
CO	168.6, C	
Cα	41.5, CH_2	3.81, dd (17.8, 4.3)<br>4.20, dd (17.8, 3.5)
NH		6.97, m (ov.)

Position	δ_C, type	δ_H, mult. (J in Hz)
**L-Pip**		
CO	172.0, C	
Cα	48.2, CH	5.57, br s
Cβ	24.4, CH_2	1.84, m (ov.)<br>2.14, m (ov.)
Cγ	20.9, CH_2	1.51, m (ov.)
Cδ	46.5, CH_2	2.66, m (ov.)<br>3.02, br d (11.5)
NH		4.94, br d (12.5)
***N*-OH-D-Ala**		
CO	169.3, C	
Cα	53.4, CH	5.07, br m
Cβ	13.5, CH_3	1.24, d (6.4)
NOH		10.29, s
**Polyketide**		
1	177.2, C	
2	75.6, C	
3	98.8, C	
4	27.0, CH_2	1.53, m (ov.)<br>1.61, m (ov.)
5	23.7, CH_2	1.32, m (ov.)<br>1.62, m (ov.)
6	41.1, CH	0.99, m (ov.)
7	70.0, CH	3.54, dq (8.0, 6.3)
8	20.2, CH_3	1.24, s
9	31.2, CH_2	0.95, m (ov.)<br>1.32, m (ov.)
10	25.7, CH_2	1.15, m (ov.)<br>1.32, m (ov.)
11	29.1, CH_2	1.23, m (ov.)
12	31.2, CH_2	1.24, m (ov.)
13	22.1, CH_2	1.26, m (ov.)
14	14.0, CH_3	0.86, t (6.3)

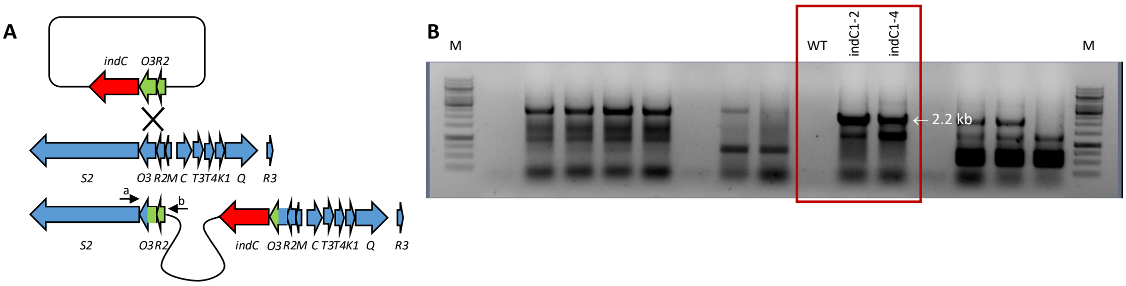

Figure S1. Generation of CS113R-indC1 strain. (A) Graphical representation of the generation of the recombinant strain; (B) PCR analysis of CS113R-indC1 mutant strain (lanes framed in red). PCR products from the wild type (WT) strain and from the CS113R-indC1 mutant strain (indC1) using oligonucleotides 113C1orf18-indC-comp-R (a) and M13-F (b). indC1-2 and indC1-4 correspond to two independent mutant strains. M, 1 kb ladder. *indC*, indigoidine reporter gene.

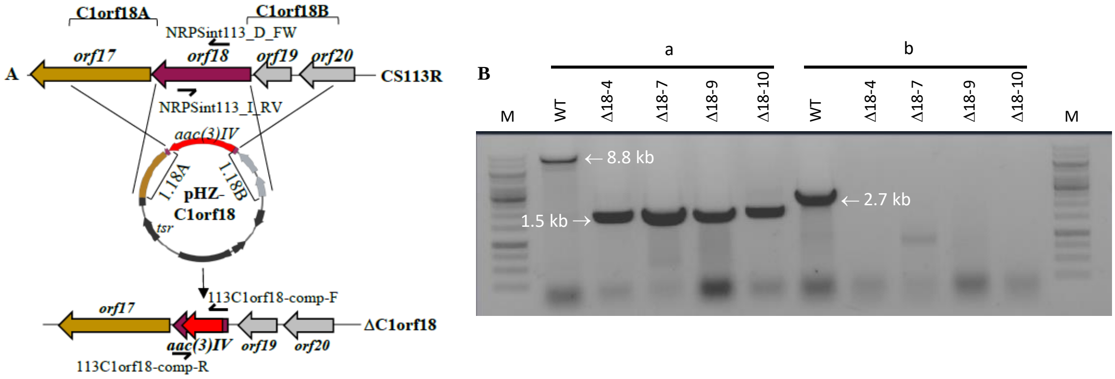

Figure S2. Generation of CS113R-ΔC1orf18 mutant. (A) Graphical representation of the generation of the mutant strain. *Orf17*, *orf18*, *orf19* and *orf20* correspond to *dpnS3*, *dpnS2*, *dpnO3* and *dpnR2*, respectively; (B) PCR analysis of CS113R-ΔC1orf18 mutant strain. PCR products from the wild type (WT) strain and from CS113R-ΔC1orf18 mutant strain (Δ18) using oligonucleotides 113C1orf18-comp-F and 113C1orf18-comp-R (a); and NRPSint113_I_RV and NRPSint113_D_FW (b). Δ18-4, Δ18-7, Δ18-9 and Δ18-10 correspond to four independent mutants. M, 1 kb ladder. *aac(3)IV*, apramycin resistance gene.

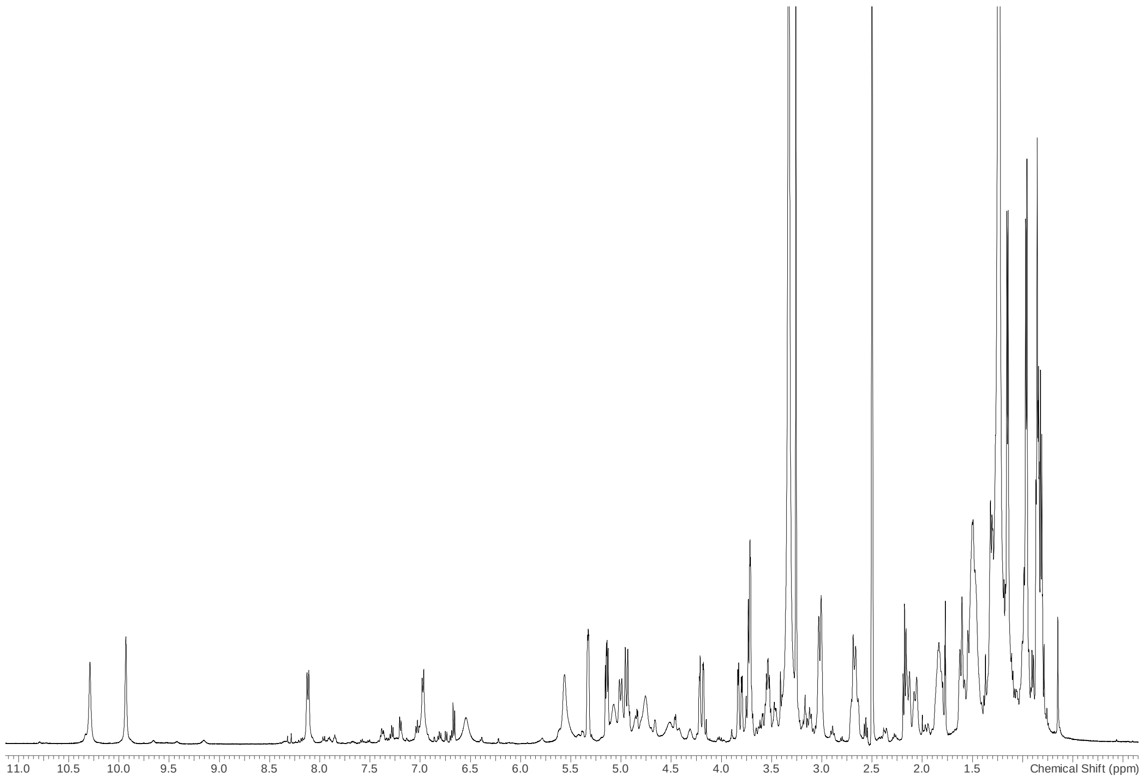

Figure S3. ^{1}H NMR spectrum of diperamycin (DMSO-$_{d6}$, 500 MHz, 24 °C).

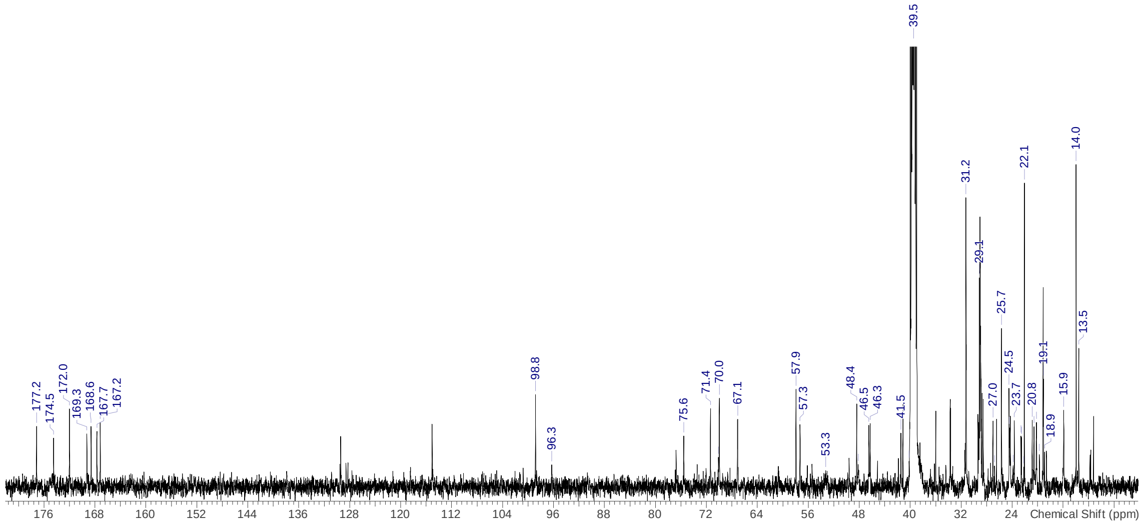

Figure S4. ^{13}C NMR spectrum of diperamycin (DMSO-d_6, 125 MHz, 24 °C).

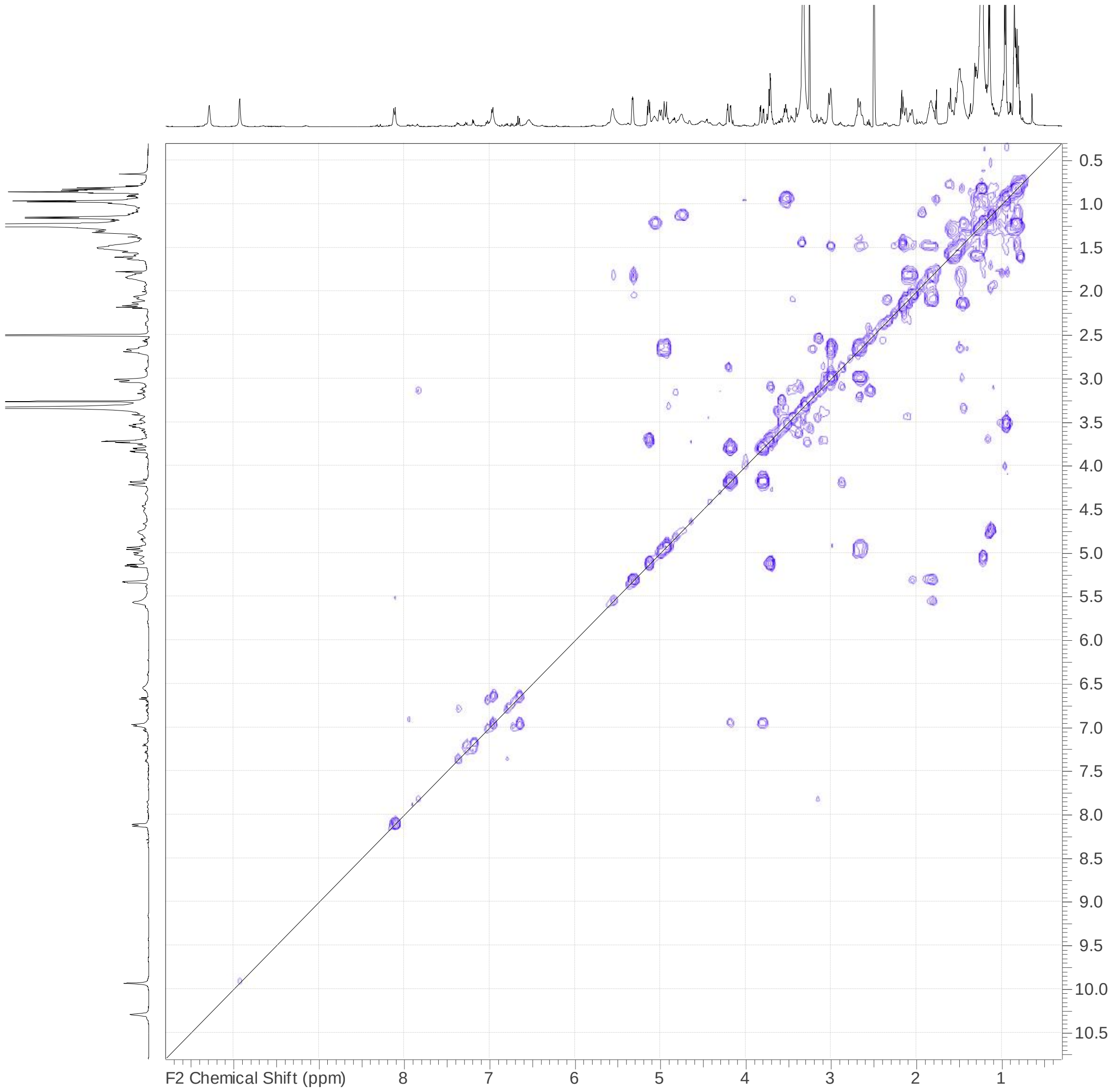

Figure S5. COSY spectrum of diperamycin.

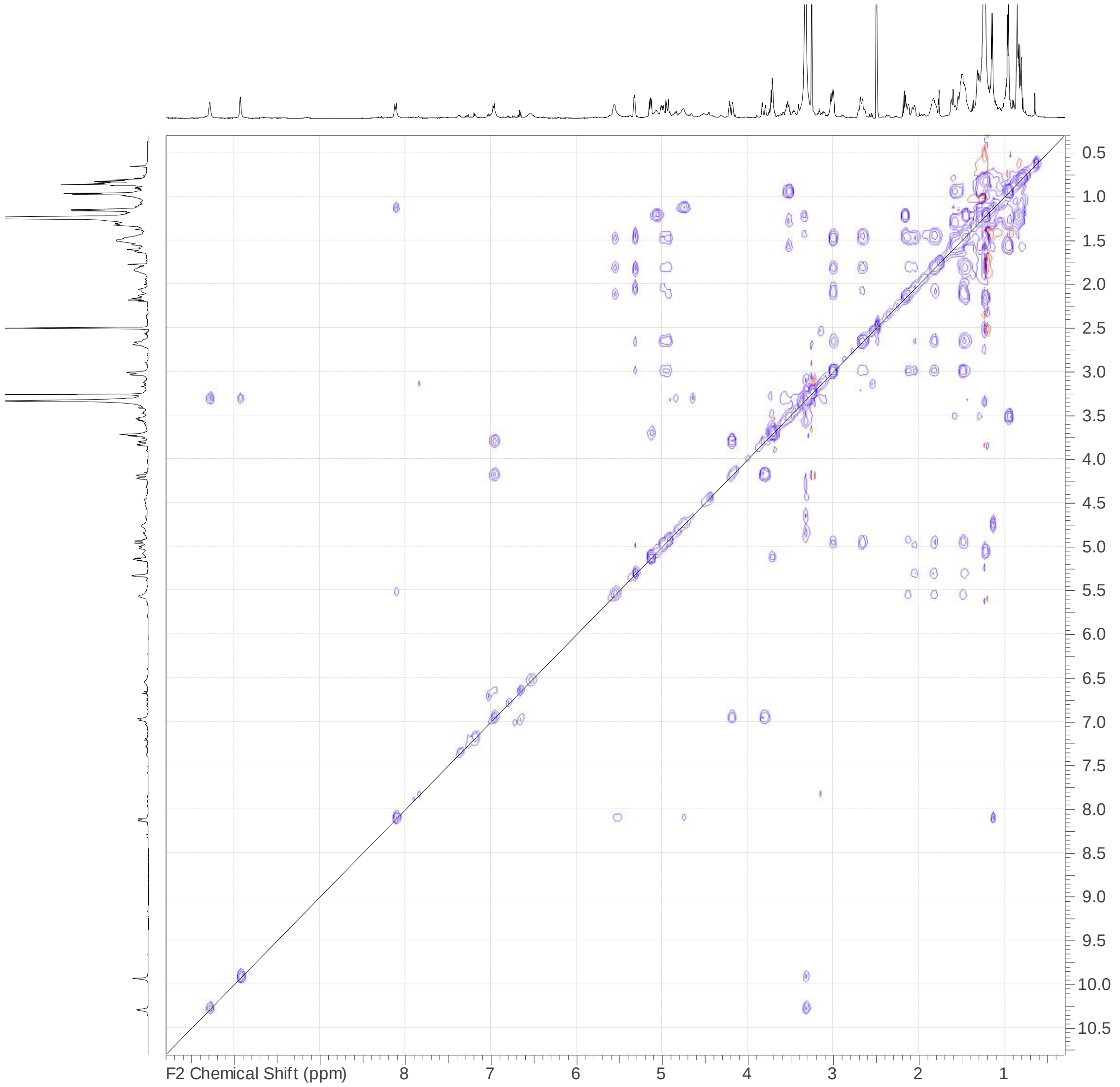

Figure S6. TOCSY spectrum of diperamycin.

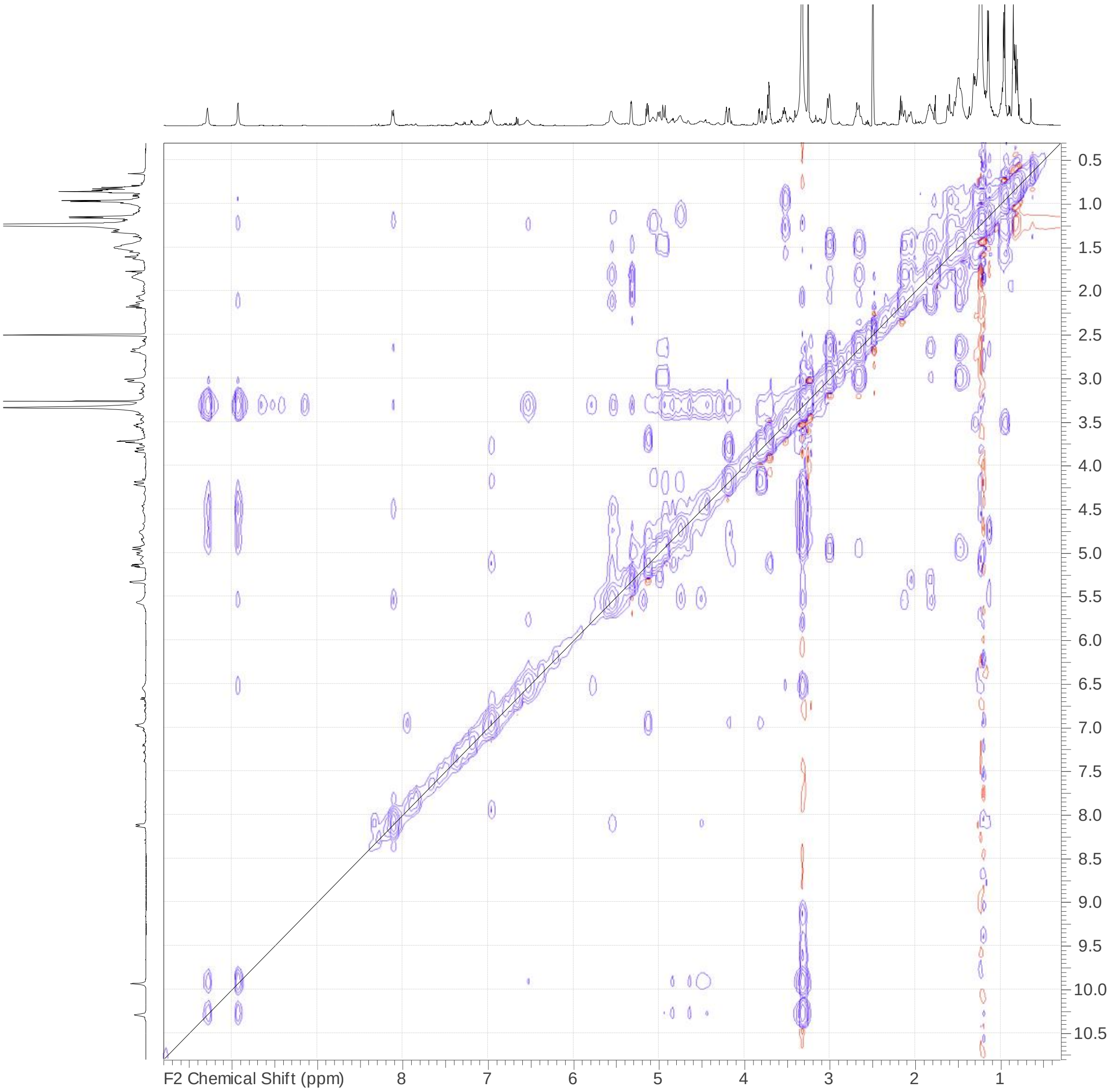

Figure S7. NOESY spectrum of diperamycin.

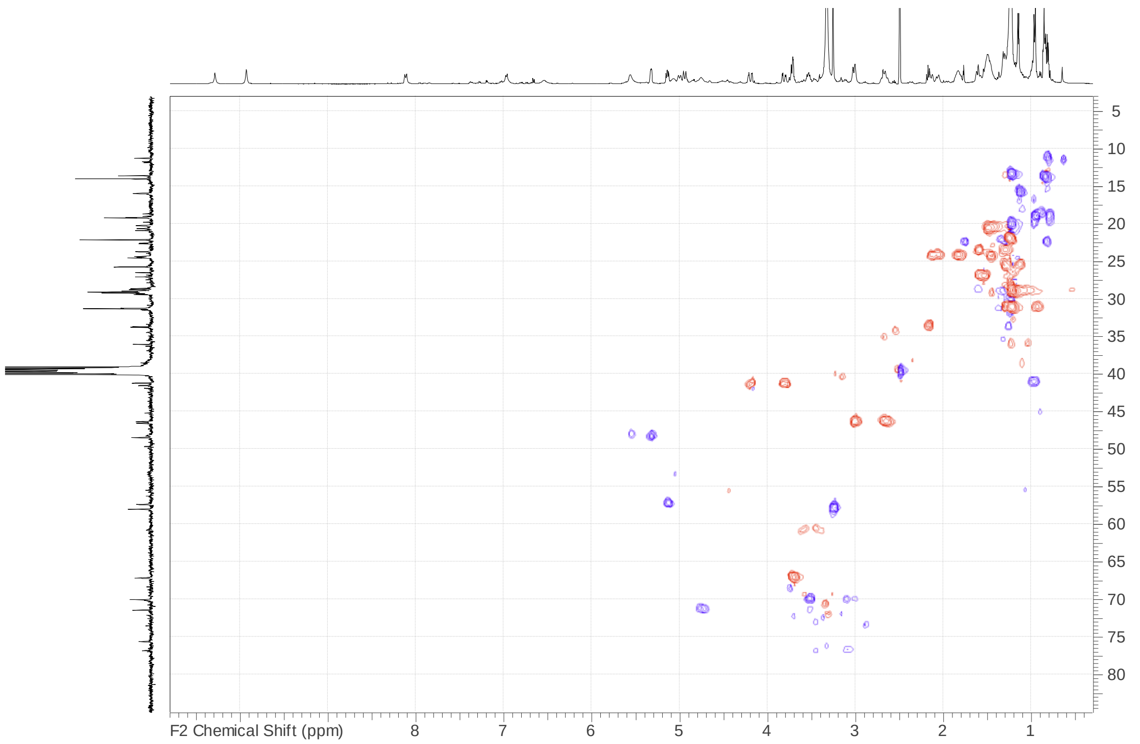

Figure S8. Multiplicity-edited HSQC spectrum of diperamycin.

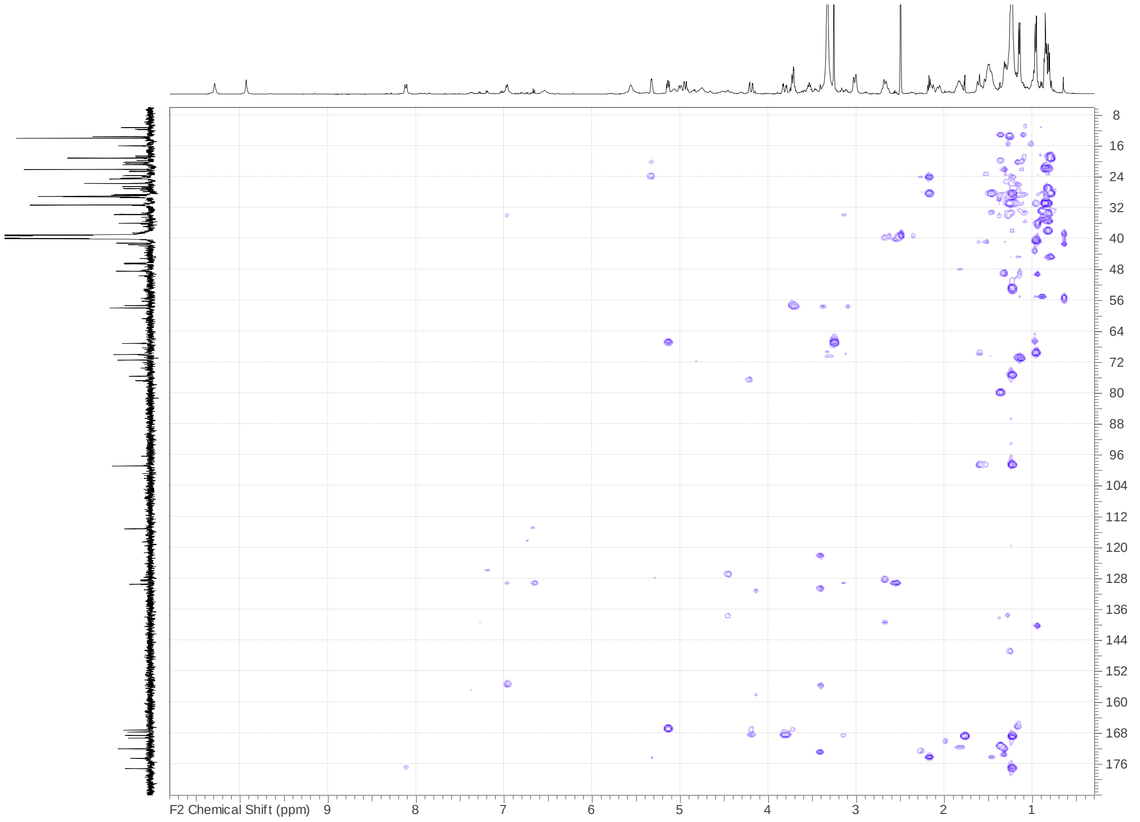

Figure S9. HMBC spectrum of diperamycin

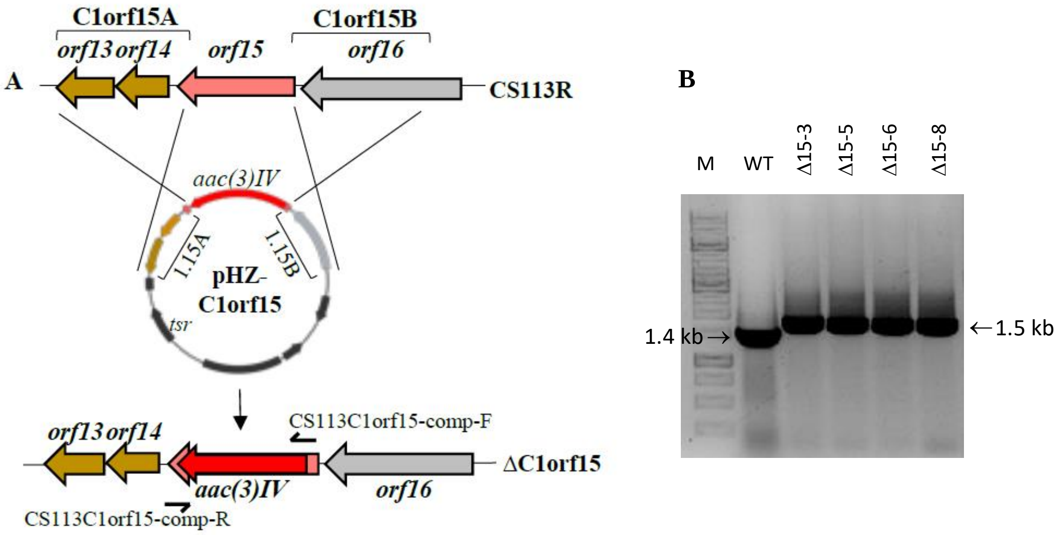

Figure S10. Generation of CS113R-ΔC1orf15 mutant. (A) Graphical representation of the generation of the mutant strain. *Orf13*, *orf14*, *orf15* and *orf16* correspond to *dpnT2*, *dpnB2*, *dpnO2* and *dpnS4*, respectively; (B) PCR analysis of CS113R-ΔC1orf15 mutant strain. PCR products from the wild type (WT) strain and from the CS113R-ΔC1orf15 mutant strain (Δ15) using oligonucleotides 113C1orf15-comp-F and 113C1orf15-comp-R. Δ15-3, Δ15-5, Δ15-6 and Δ15-8 correspond to four independent mutants. M, 1 kb ladder. *aac(3)IV*, apramycin resistance gene.

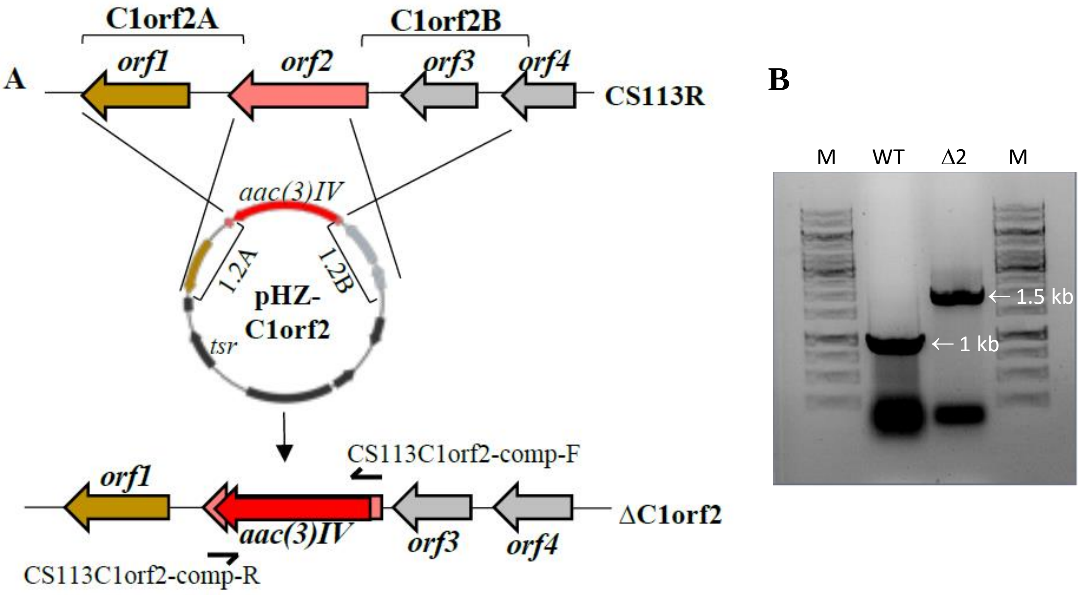

Figure S11. Generation of CS113R-ΔC1orf2 mutant. (A) Graphical representation of the generation of the mutant strain. *Orf1*, *orf2*, *orf3* and *orf4* correspond to *dpnL*, *dpnZ*, *dpnA* and *dpnB1*, respectively; (B) PCR analysis of CS113R-ΔC1orf2 mutant strain. PCR products from the wild type (WT) strain and from the CS113R-ΔC1orf2 mutant strain (Δ2) using oligonucleotides 113C1orf2-comp-F and 113C1orf2-comp-R. M, 1 kb ladder. *aac(3)IV*, apramycin resistance gene.

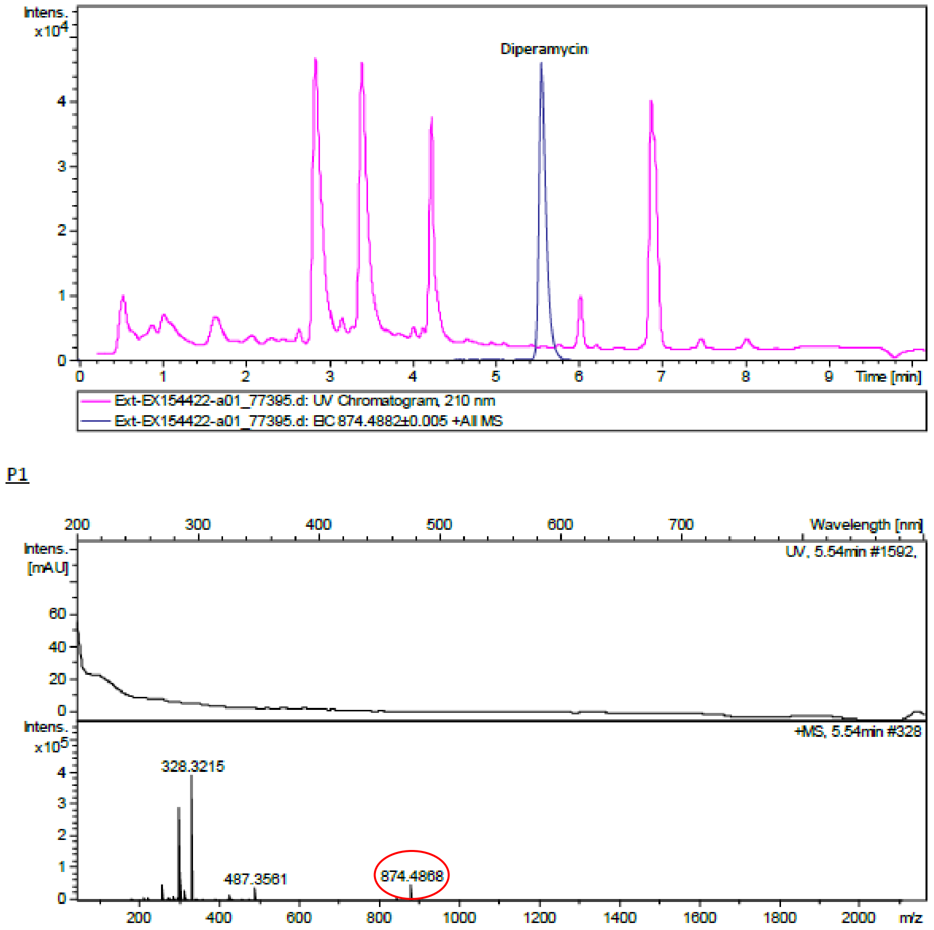

Figure S12. Complementation of CS113R-ΔC1orf2 mutant with pSETETc-C1orf2. Chromatogram at 210 nm and extracted ion ($[M+NH_4]^+$) chromatogram and HRMS spectrum of diperamycin.
